# Red Blood Cell Transfusion Strategies in Cardiovascular Interventions

**DOI:** 10.31083/j.rcm2507252

**Published:** 2024-07-08

**Authors:** Melissa Foglietta, Elena Bacigalupi, Francesco Radico, Jacopo Pizzicannella, Marianna Appignani, Federica De Donno, Patrizia Di Gregorio, Francesco Pelliccia, Marco Zimarino

**Affiliations:** ^1^Department of Neuroscience, Imaging and Clinical Sciences, University “G. d’ Annunzio” Chieti-Pescara, 66100 Chieti, Italy; ^2^Cardiology Department, “SS. Annunziata” Hospital, 66100 Chieti, Italy; ^3^Department of Engineering and Geology, University “G. D’Annunzio” Chieti-Pescara, 65127 Pescara, Italy; ^4^Transfusion Medicine Department, “SS. Annunziata” Hospital, 66100 Chieti, Italy; ^5^Department of Cardiovascular Sciences, Sapienza University, 00185 Rome, Italy

**Keywords:** red blood cell transfusion, acute coronary syndrome, cardiac surgery, structural interventions

## Abstract

Acute coronary syndrome, cardiac surgery, and cardiac structural interventions are among the most common situations leading to allogeneic red blood cell consumption due to the prevalence of bleeding and anemia. The wide variability in the use of transfusions derives from the current lack of data, and the absence of strong evidence and clear guideline recommendations. The current approach is to avoid unnecessary blood transfusions and limit their use to life-saving conditions; this conservative strategy derives from often controversial and inconclusive results of observational and randomized studies where liberal and restricted red blood transfusion strategies seemed to have similar outcomes. The pivotal question for future research lies in elucidating whether blood transfusions function as an active participant or merely a catalyst in amplifying adverse events. The present review aims to summarize the current literature data and critically analyze the available evidence for red blood transfusions in cardiac interventions.

## 1. Introduction 

Ischemic heart disease is the major responsible factor of cardiovascular deaths [1]. Over the last few decades a huge interest has grown around valvular heart disease, due to the increase in life expectancy and the availability of minimally invasive treatments [2]. In both scenarios, transcatheter management—combined with the use of antithrombotic therapies—has lowered the risk of untoward events, and at the same time has also been associated with a significant increase in bleeding, and subsequent need for red blood cell (RBC) transfusions, due to the prevalence of anemia and the complexity and fragility of such patients.

Annually, more than 100 million units of blood are collected worldwide, and RBC transfusions are one of the most overused procedures in hospitals. Anemia can lead to tissue hypoxia in cardiovascular patients [3]; on the other hand, the use of allogeneic RBC transfusion—still high in cardiac interventions—is associated with adverse short-term and long-lasting effects [4, 5]. Therefore, reducing the unnecessary use of RBC transfusions is important both for patient outcomes and to optimize the use of a limited resource. Safe RBC transfusion practices depend on the match between the right indication, the right amount of RBC transfusions and the right timing.

The definition of the optimal hemoglobin (Hb) threshold for RBC transfusion in the context of acute coronary syndrome (ACS) and transcatheter procedures or cardiac surgery is an unmet clinical need and most of the information derive from observational studies and few randomized controlled trials (RCTs) [6, 7].

Beyond the uncontested value of RBC transfusion in life-threatening bleeding, in many cases there is minor evidence about its benefit use with wide variability in transfusion decision-making due to the absence of any Hb thresholds to guide clinicians [8]. Frequently, RBC transfusion is used as a simple solution for fluid repletion or to correct a lower-than-expected Hb value even if the true rationale of the use is to reduce tissue hypoxia—an important risk factor in patients at high preoperative risk-related to an increased risk of complications and death [9].

Therefore, if theoretically, RBC transfusions increase the levels of Hb, whilst improving both tissue oxygenation and oxygen-carrying capacity, unfortunately at the same time, the administration of this blood could also result in heart failure from fluid overload, infection, and inflammation. In addition, immuno-mediated reactions, and changes in deformability of stored RBC or Hb oxygen affinity [10] could justify the impaired outcomes such as the augmented incidence of acute kidney injury and the paradoxical increase of short-term mortality in cardiac patients receiving RBC transfusion [11, 12].

The relative or absolute role of blood transfusions in determining poor outcomes is questioned in favor of a patient-centered condition; greater disease burden, malnutrition, chronic anemia and diseases, subclinical bleeding diathesis, coagulopathy, peripheral vascular disease, chronic kidney disease, and fragility. These factors identify patients who will likely receive a RBC transfusion and therefore the key question is still open whether it is a marker or a mediator of impaired outcome.

The present review aims to analyze the use of RBC transfusions in various cardiovascular settings and tries to define the clinical risk-benefit ratio to guide clinicians in high-risk scenarios.

## 2. The Currently Available Recommendations on Blood Transfusion

The Association for the Advancement of Blood & Biotherapies (AABB) recently issued international guidelines on RBC transfusion. The panel of experts used a systemic review and a meta-analysis from Cochrane to determine optimal transfusion strategies. For adult populations, the experts reviewed 45 RCT—with a total of 20,599 patients [13]—including the expanded evidence from numerous trials that emerged after the publication of 2018 AABB guidelines [14] and compared the patient outcomes with restrictive Hb-based transfusion thresholds—defined as 7 to 8 g/dL—with more liberal transfusion thresholds—defined as 9 to 10 g/dL— and reexamined the transfusion threshold evidence.

Moreover, for adults, very frequent subgroups in clinical practice—such as preexisting coronary disease, cardiac surgery, orthopedic surgery, and oncologic or hematological conditions—were considered to examine the harm and the benefit of a particular RBC transfusion threshold.

Analyzing the 45 RCTs with adult participants selected by the authors—8 concerning cardiac surgery, 8 critical care, and 3 acute myocardial infarction (MI)—the authors agreed that the most common liberal Hb transfusion threshold was 9 to 10 g/dL, and the most common restrictive threshold was 7 to 8 g/dL. The rationale for recommending such “magic numbers” —7 g/dL of Hb—has no clinical or biological basis for expecting different outcomes between 7 and 8 g/dL but the authors simply followed the trials’ strategies.

Current guidelines considering the category of hospitalized and hemodynamically stable adult patients without hematological and oncological disorders, recommended—with a moderate level of evidence—a restrictive transfusion strategy considering RBC transfusion for Hb concentration <7 g/dL. However, in accordance with the largest trials, the authors agree the adoption of modulation of such a “restrictive” strategy using a Hb threshold of 7.5 g/dL may be adopted for cardiac surgery [15] and 8 g/dL for patients with preexisting cardiovascular disease. The expert panel refrained from providing a recommendation for the transfusion threshold in patients with acute myocardial infarction, due to the imprecision of available data at the time of the issue [16], since the Myocardial Ischemia and Transfusion (MINT) trials results were available later on [17].

## 3. Acute Coronary Syndromes

Anemia in patients hospitalized for acute coronary syndrome is common and associated with an increased risk of in-hospital mortality [18, 19]. From a physiopathological perspective, RBC transfusion might mitigate ischemic injury by enhancing oxygen supply to myocardial tissues, potentially reducing the risk of reinfarction or mortality. The 2014 American Heart Association (AHA) guidelines [20] advised against routine blood transfusion for stable ACS patients with Hb levels above 8 g/dL, while the 2023 European Society of Cardiology (ESC) guidelines [21] refrained from formally recommending an optimal transfusion strategy for ACS due to insufficient clinical trial data.

Previous observational data suggest that the employment of a liberal RBC transfusion approach might elevate the risk of short- and long-term mortality, along with myocardial reinfarction [22]. The few RCTs [15, 23, 24] comparing Hb-guided restrictive versus liberal transfusion strategies in ACS have limited long-term outcome data and gave inconsistent findings. In the Restrictive and Liberal Transfusion Strategies in Patients With Acute Myocardial Infarction (REALITY) trial [25], both groups exhibited similar occurrences of the composite outcome at 30 days, meeting the non-inferiority criteria (11% restrictive vs 14% liberal, relative risk (RR) 0.79 [1-sided 97.5% confidence interval (CI) 0.00–1.19]). However, the liberal transfusion strategy group showed numerically higher values for all adverse events of the composite endpoint, without sufficient power to establish the superiority of the restrictive strategy (upper bound of 1-sided 97.5% CI >1.00).

Interestingly, the pre-planned 1-year follow-up revealed conflicting results. The restrictive strategy failed to demonstrate its non-inferiority regarding major adverse cardiovascular events (MACE) (35% restrictive vs 28% liberal, RR 1.13 [1-sided 97.5% CI 0–1.43]), and showed a higher incidence of events, including all-cause mortality.

The recent open-label randomized MINT trial [17] represents the largest randomized controlled study comparing conservative versus liberal RBC transfusion strategies among hospitalized ACS patients. A total of 3504 patients identified with MI and Hb <10 g/dL were randomized to a restrictive-strategy group—transfusion permitted but not required for Hb <8 g/dL and strongly recommended for Hb <7 g/dL or for recurrent angina—or to a liberal-strategy group; in the trial, 1 RBC unit was administered after randomization and then transfused to maintain Hb >10 g/dL until the time of hospital discharge or for 30 days. The primary outcome was a composite outcome of death or MI at 30 days. The trial revealed no significant differences between the two approaches in the primary endpoint and suggested a trend towards a benefit in the reduction of the primary endpoint for the liberal-strategy group compared to the restrictive transfusion strategy group (14.5 vs 16.9%, *p* = 0.07). Cardiac death was more common in the restrictive—rather than in the liberal-strategy group (5.5% and 3.2%, respectively; RR 1.74; 95% CI, 1.26–2.40). A marginal benefit for the liberal strategy was consistently documented for death, recurrent MI, ischemia-driven unscheduled coronary revascularization, or hospital readmission. Among the patients with type 1 MI, the restrictive transfusion strategy led to more primary-outcome events than the liberal-strategy (RR 1.32; 95% CI, 1.04–1.67), with no apparent effect among the patients with type 2 MI (RR 1.05; 95% CI, 0.85–1.29).

The promising findings from the liberal arm of MINT presented a new perspective on ACS patient management compared to previous transfusion trials across diverse patient populations and treatments. These results were not adopted to formulate current recommendations and to support this limited resource as an intervention in all patients with anemia following MI.

## 4. RBC Transfusion in Cardiac Surgery 

Cardiac surgery carries a high risk of perioperative complications; among these, bleeding and postoperative anemia are very common [26, 27].

Perioperative bleeding is multifactorial, being triggered by drugs, hemodilution, and hemolysis induced by cardiopulmonary bypass itself or by suboptimal surgical hemostasis [28, 29]. Moreover, patients undergoing cardiac surgery have multiple comorbidities that may increase the risk of bleeding and further complications [30].

Ideally, the Hb level that prevents tissue hypoxia should guide RBC transfusion. Determining the specific Hb level at which an RBC transfusion should be undertaken has proven difficult [9], and studies have focused on lowering the threshold Hb for transfusing RBCs to rationalize the administration—considering the reduction in the availability of blood products—and to reduce the risks associated with transfusion.

Moreover, although direct transmission of infectious agents via allogeneic RBC transfusion is quite low in developed countries, blood transfusion is associated with immunomodulation which may affect infection risk [31].

Evidence supporting the use of restrictive transfusion strategies in cardiac surgery patients has been growing in recent years. However, findings have been controversial, with some studies documenting the safety of a restrictive strategy in cardiac surgery, while others raise concerns.

The two largest RCTs comparing RBC transfusion restrictive strategies found no differences in the primary outcomes. For these, the parameters for Hb were <7.5g/dL—to liberal—for Hb <9.0 g/dL in the Transfusion Indication Threshold Reduction (TITRe2) trial [32] and Hb <9.5g/dL in the Transfusion Requirements in Cardiac Surgery (TRICS) trial [9]. The TITRe2 study documented no differences at 3 months in the primary composite outcome; however, this study demonstrated higher 3-month mortality in the restrictive group (4.2%) compared to the liberal one (2.6%). Further analyses of the TITRe2 study showed no advantages in terms of cost-effectiveness when comparing restrictive and liberal RBC transfusion practices—a part of the inevitable difference related to the transfusion themselves. Similarly, the TRICS study found similar primary composite outcomes and mortality rates at 28 days. Recent meta-analyses [33]—including these studies confirmed no differences between these strategies. Overall, the take-home message seems that “one size fits all” is unlikely in cardiac surgery, considering the heterogeneity of cardiac surgery patients and factors influencing the perioperative period. Hence, a more personalized approach is warranted and awaited [34], highlighting the importance of carefully assessing each patient’s risk factors and clinical situation when deciding on the appropriate transfusion strategy [35]. This view of optimization for RBC transfusions should be pursued through the implementation of local protocols [36].

## 5. RBC Transfusion in Structural Interventions

The advent of transcatheter approaches for non-coronary cardiac disease—mainly valvular repair or replacement—has provided a viable alternative to cardiac surgery in patients with risk factors or clinical conditions that render them ineligible for surgery; however, bleeding is a common complication during structural heart interventions, due to various factors: access-site vascular complications are frequent, mainly due to the use of large sheath size catheters, antithrombotic therapy is required due to the deployment of intracardiac or intravascular devices, and this is complicated by the fact that patients undergoing these procedures typically have a high intrinsic bleeding risk linked to frailty, advanced age or comorbidities [37, 38].

In an early meta-analysis [39], including more than 3500 patients undergoing transcatheter aortic valve implantation (TAVI), the rate of any bleeding amounted to 41% with a pooled rate of life-threatening and major bleeding of 15.6% and 22.3% respectively. Transfusions were recorded only in a small proportion of the included studies, with a pooled rate of administration of at least one RBC unit for 46% of patients. Kolte *et al*. [40] analyzied the National Inpatient Sample database, and reported a rate of RBC transfusions of 17.3%, among 46,710 TAVIs performed from 2012 to 2015 in the United States. Interestingly RBC transfusion rates decreased significantly from 29.5% during the first quarter of 2012 to 10.8% during the third quarter of 2015 (*p* < 0.001). Most recently, in the PARTNER-3 trial, a very low rate of transfusion (2.0% at 30 days) was noted for low-risk patients undergoing transfemoral TAVI with third-generation systems [41]. In patients undergoing mitral valve transcatheter edge-to-edge repair (M-TEER), Körber *et al*. [42] reported an incidence of any bleeding of 21.6% (7.4% major). Similarly, in the COAPT trial, the rate of bleeding was 12.6%, and 4.3% for major or life-threatening bleeding [43].

In M-TEER patients, major or life-threatening bleeding has been reported as an independent predictor of cardiovascular death, while minor bleeding was unrelated to hard outcomes [43].

Therefore, RBC transfusion is frequently administered empirically post transcatheter interventions. However, consensus regarding the relative benefits and optimal transfusion strategy [44] remains elusive, as its utilization appears controversial, supported by evidence—suggesting independent associations with worse outcomes following TAVI, regardless of Hb concentrations or drop [45]. Whether RBC transfusion per se represents an independent predictor of outcomes still needs to be clarified and future research efforts are required.

In the specific setting of transfemoral TAVI, explored in The Transfusion Requirements in Transcatheter Aortic Valve Implantation (TRITAVI) registry, we highlighted higher cardiovascular risk in transfused patients as compared to matched non-transfused ones [46, 47]. In this large, multicentric study, 2587 patients were enrolled, and RBC transfusion was administered in 421 cases (16%). After propensity-matching, 842 patients were identified, RBC transfusion was associated with increased mortality (hazard ratio, 2.07 [95% CI, 1.06–4.05]; *p* = 0.034) and acute kidney injury (hazard ratio, 4.35 [95% CI, 2.21–8.55]; *p* < 0.001). In the multivariable Cox proportional hazards regression model, RBC transfusion independently correlated with 30-day mortality, irrespective of major vascular complications and major bleeding.

Confirming these data, Kolte *et al*. [40] compared 7995 pairs of propensity-matched transfused and non-transfused TAVI patients, and found that RBC transfusion was associated with an increased risk of in-hospital mortality, infection, and transient ischemic attack/stroke in patients without bleeding but not in those with overt bleeding.

So, RBC transfusion should be cautiously used in TAVI patients, although its utility and optimal threshold—especially among patients with overt bleeding—warrants further prospective investigation.

## 6. Strategies to Avoid RBC Transfusion

Over time, the progressive advancements in procedural techniques, alongside the reduction in peri-procedural complications—such as vascular peri-operative issues—and concurrent improvement of results in transcatheter procedures with the latest prostheses, coupled with changes in perioperative antithrombotic treatments, have provoked an extreme decrease in numbers of RBC transfusions.

In percutaneous coronary interventions, the shift adapted more than a decade ago towards the systematic use of radial as opposed to femoral access, which dramatically reduced the bleeding risk [48]; more complex procedures still require more aggressive antithrombotic therapies, but procedure optimization may contain the duration of antiplatelet therapy and therefore can contain the bleeding risk [49].

In structural interventions, the smaller introducer sheath sizes, the increasing use of transfemoral or endovascular access as opposed to transapical access, the advanced generations of devices, the greater operator experience, the better patient selection, and, the extension of the indications to younger patients with lower baseline risk have contributed to the current reduction of bleeding and RBC transfusion rates [40]. To confirm this trend, several practices could be implemented before—identifying peri-operative risk factors, during, and after the procedures. The extension of structural intervention to centers without on-site cardiac surgery might reverse this process [50].

Older age, female sex, small-size people, presence of peripheral vascular disease, or chronic kidney disease are all known risk factors for vascular and bleeding complications that can be assessed and valued priorly [4].

Moreover anemia—with a prevalence of 60%—and coagulopathy at baseline—as indicators of frailty and frequently associated with valvulopathies or coronary syndrome—are clearly the largest predictor of blood transfusion.

Iron supplementation or epoetin treatment, a full nutritional assessment, and a strict pre-treatment protocol to detect tractable causes of the anemia in selected patients—such as in women population who often have baseline anemia and require RBC transfusion [47, 51]—might increase baseline Hb levels, although such preventive measures failed to improve outcomes.

In addition, in percutaneous procedures, the avoidance of vascular complications achieved through prior careful access site selection can limit the necessity of RBC transfusion; during procedures, the better use of techniques for access and closure [52] and a controlled antithrombotic therapy—activated clotting time-guided procedures—may reduce the risk of bleeding for the vascular access and nonaccess sites. After the procedure, changes in post-operative antithrombotic treatment—such as a single instead of dual antiplatelet therapy or single anticoagulant therapy in case of concomitant indication for oral anticoagulation—have been associated with improved safety and so are largely and clearly recommended as strategies to avoid RBC transfusion use [53].

A further word of caution is needed for the management of concurrent coronary artery disease (CAD) in patients undergoing TAVI, since antiplatelet therapy increases the risk of bleeding. Current evidence, although derived from observational studies, suggest the deferral of percutaneous coronary intervention (PCI) after TAVI in order to contain adverse events that are clustered during the concurrent treatment of both pathologies [54, 55].

## 7. Conclusions

Observational and randomized trials contribute to guiding clinicians to adopt restrictive strategies and not consider RBC transfusion as a solution for fluid repletion or to correct a lower-than-expected Hb value. Identifying RBC transfusion as an independent predictor of early mortality and poor outcomes in cardiac interventions, trial results promote the avoidance of unnecessary blood transfusion and limit them as much as possible to limit the deleterious effects. Therefore, optimal and safe transfusion thresholds and protocols are mandatory to guide restrictive strategies. Liberal strategies could have partial benefits in anemic patients with acute MI, but additional studies would be needed to add evidence on to the optimal RBC transfusion strategy and to also clarify the currently limited data on long-term outcomes.

## Abbreviations

AABB, Association for the Advancement of Blood & Biotherapies; ACS, acute coronary syndrome; Hb, hemoglobin; MI, myocardial infarction; M-TEER, mitral valve transcatheter edge-to-edge repair; RBC, red blood cells; RCT, randomized controlled trial; TAVI, transcatheter aortic valve implantation.
